# Using mindfulness-based intervention to promote executive function in young children: a multivariable and multiscale sample entropy study

**DOI:** 10.1093/cercor/bhae330

**Published:** 2024-09-05

**Authors:** Sha Xie, Shuqi Lu, Jiahao Lu, Chaohui Gong, Chunqi Chang

**Affiliations:** Faculty of Education, Shenzhen University, Shenzhen 518055, China; School of Biomedical Engineering, Shenzhen University Medical School, Shenzhen University, Lihu Campus, Shenzhen 518055, China; School of Biomedical Engineering, Shenzhen University Medical School, Shenzhen University, Lihu Campus, Shenzhen 518055, China; School of Biomedical Engineering, Shenzhen University Medical School, Shenzhen University, Lihu Campus, Shenzhen 518055, China; School of Biomedical Engineering, Shenzhen University Medical School, Shenzhen University, Lihu Campus, Shenzhen 518055, China; Peng Cheng Laboratory, Shenzhen 518055, China

**Keywords:** brain complexity, multivariable and multiscale sample entropy, executive function, mindfulness-based intervention

## Abstract

Early childhood marks a pivotal period in the maturation of executive function, the cognitive ability to consciously regulate actions and thoughts. Mindfulness-based interventions have shown promise in bolstering executive function in children. This study used the functional near-infrared spectroscopy technique to explore the impact of mindfulness-based training on young children. Brain imaging data were collected from 68 children (41 boys, aged 61.8 ± 10.7 months) who were randomly assigned to either an intervention group (*N* = 37, aged 60.03 ± 11.14 months) or a control group (*N* = 31, aged 59.99 ± 10.89 months). Multivariate and multiscale sample entropy analyses were used. The results showed that: (1) brain complexity was reduced in the intervention group after receiving the mindfulness-based intervention in all three executive function tasks (*p*s < 0.05), indicating a more efficient neural processing mechanism after the intervention; (2) difference comparisons between the intervention and control groups showed significant differences in relevant brain regions during cognitive shifting (left dorsolateral prefrontal cortex and medial prefrontal cortex) and working memory tasks (left dorsolateral prefrontal cortex), which corroborates with improved behavioral results in the intervention group (*Z* = −3.674, *P* < 0.001 for cognitive shifting; *Z* = 2.594, *P* < 0.01 for working memory). These findings improve our understanding of early brain development in young children and highlight the neural mechanisms by which mindfulness-based interventions affect executive function. Implications for early intervention to promote young children’s brain development are also addressed.

## Introduction

Executive function (EF) encapsulates the cognitive prowess essential for the deliberate regulation of one’s actions and thoughts, representing a sophisticated mental process (Zelazo and Müller 2002; Griffin et al. 2016). Recognized as integral to children’s preparatory cognitive abilities for formal education (Blair 2002; Guedes et al. 2023), early childhood EF undergoes pivotal developmental stages, playing a foundational role in behavior regulation (Moriguchi and Hiraki 2011). The cultivation of EF during this critical period lays the groundwork for subsequent advancements in children’s social, emotional, and cognitive competencies (Griffin et al. 2016).

Empirical evidence underscores the potential of mindfulness training in significantly enhancing EF among young children and simultaneously mitigating problematic behavioral manifestations (Flook et al. 2015; Razza et al. 2015). Despite this promising evidence, existing studies often provide a limited examination of the tripartite components of EF—working memory (WM), cognitive flexibility (CF), and inhibitory control (IC). Moreover, there is a paucity of research that substantiates the efficacy of these interventions through neuroimaging evidence, leaving a critical gap in understanding the neural mechanisms underlying these improvements.

To address this gap, our study aims to provide a comprehensive exploration of the effects of a mindfulness-based intervention on EF in young children. By employing multivariable and multiscale sample entropy analysis, we seek to elucidate the changes in brain complexity associated with enhanced EF. This approach not only advances the current literature by integrating detailed neuroimaging evidence but also offers novel insights into how mindfulness training can be effectively utilized to promote cognitive development in early childhood.

### EF and its three components

EF is commonly divided into three cores: IC (covering self-control, such as behavioral inhibition, and interference control, such as selective attention and cognitive inhibition), WM (ability to temporarily store and manipulate information), and CF (the ability to flexibly shift the focus of one’s mental frame) (Blair 2016; Moriguchi 2017). Numerous studies have noted significant activation of the prefrontal cortex of the brain when performing EF-related cognitive tasks (Huttenlocher and Dabholkar 1997; Scheibel and Levin 1997; Anderson 2008). During infancy and preschool, these core components of executive functioning develop, forming a critical foundation that sets the stage for the development of higher cognitive processes in adulthood. A series of studies in structural equation modeling have shown that each subdomain of EF plays a different role in performance on a range of executive outcome metrics, underscoring the importance of recognizing the diversity of these sub-processes (Garon et al. 2008). Functional near-infrared spectroscopy (fNIRS) measures task-related changes in cerebral hemodynamics. Because of its portability, fNIRS is widely used in the research of EF. Therefore, the present study used the fNIRS technique to measure preschoolers’ task performance in three different EF cores: IC (Cragg and Nation 2008; Inoue et al. 2012), CF (Davidson et al. 2006), and WM (Baddeley and Hitch 1994; Smith and Jonides 1999).

### Advances in studying the EF’s three components

CF, one of the primary EFs, typically begins to develop at approximately 3–4 years of age. During the first 2 years of life, early CF skills are primarily supported by attentional control and language skills, both of which are critical to the early development of goal-directed behavior (Hendry et al. 2016). Research suggests that IC and WM underlie CF development, beginning at approximately 3 years of age, and that they each contribute to successful switching behaviors in different ways (Blakey et al. 2016). In particular, IC promotes CF by allowing children to ignore task-irrelevant information, while WM functions by allowing children to maintain and update new sequencing rules. Recent research has noted that cognitive performance is highly dependent on the task and context employed (Perry et al. 2014; Yee and Thompson-Schill 2016).

The Dimension-Converting Card Sorting Test (DCCS) is a classic task for assessing CF. Findings show that when three-year-olds are asked to sort complex stimuli, they typically sort according to an initial rule, whether that rule is based on shape or color. However, when asked to switch to a new rule, most three-year-olds failed to adapt and continued to sort according to the initial rule (Zelazo 2006). In other words, they tended to stick to their initial response pattern when confronted with a request for a rule change. In contrast, children around the age of four usually can sort stimuli according to the new rules. Thus, research on the DCCS task tends to suggest that the critical period of CF development is around three to four years of age, with the most significant developmental advances manifested in the ability to overcome perseveration (Munakata et al. 2012). Significant neural activity occurred in the bilateral inferior prefrontal region during the conduct of the experiment. Thus, developmental psychologists believe that maturation of the lateral prefrontal cortex (LPFC) may play a key role in the development of cognitive switching abilities (Davidson et al. 2006; Gupta et al. 2009).

During preschool years, children’s ability to control potentially disruptive thought processes and behaviors, namely IC ability, also develops rapidly. Three-year-olds typically have difficulty performing tasks that require IC of attentional and motor responses, such as inhibiting dominant responses to follow rules. However, by the age of five, their performance on these tasks improves significantly (Carlson 2016). Indeed, IC is thought to make an important contribution to individual differences and developmental changes in various cognitive abilities, such as attention, memory, reading comprehension, and theory of mind (Carlson et al. 2004). The Go/No-Go task is widely recognized as an important measure of IC, and the research of Moffitt et al. (2011) has shown that children from the ages of 3–11 years perform better in terms of IC. However, with the normal aging process, people’s IC declines significantly (Hasher and Zacks 1988; Hasher et al. 1991). In the field of neuroimaging, studies on response inhibition tasks have shown that such tasks persistently and specifically activate the right inferior frontal cortex. In addition, analyses of functional connectivity have found that short-range connections between the right frontal and right parietal cortex show stronger partial coherence in 4- to 6-year-old children than in adults (Mehnert et al. 2013). These findings further emphasize the uniqueness and developmental dynamics of children’s brain networks during IC.

The concept of WM originates from an in-depth understanding of short-term memory, which is primarily responsible for the temporary storage of a limited amount of information over a short period. WM is closely linked to IC, and the two are often interdependent and work together. WM declines progressively with age and gradually improves during early development. There is a close correlation between this change in WM and the decline in processing speed with age and its enhancement during early development. (Fiore et al. 2012; Fournet et al. 2012). When performing WM tasks, the frontal and parietal cortical regions show significant activation (Tsujimoto et al. 2004; Buss et al. 2014). Furthermore, there is a positive correlation between children’s age and activity, response accuracy, and speed in the LPFC (Perlman et al. 2016). These findings highlight the developmental dynamics of children’s WM capacity and its neurophysiological basis in brain networks.

### Advances in early intervention studies

EF can be improved with a targeted training (Klingberg 2010; Diamond and Lee 2011). Research has demonstrated positive outcomes from various interventions, including aerobic exercise (Bryant 2019), mindfulness (Farb et al. 2007), yoga (Gothe et al. 2014), and the Tools of the Mind early childhood program (Manjunath and Telles 2001). Notably, mindfulness-based training has been widely implemented in early childhood classrooms (Zelazo et al. 2018; Li et al. 2019; Xie et al. 2022), emphasizing the potential of mindfulness to enhance EF by reducing mind-wandering and improving focus (Hölzel et al. 2011).

Mindfulness interventions incorporate key components such as physical relaxation, breathing exercises, mental imagery, and mind–body awareness to foster an integrated mind–body connection (Thera 1972; Kabat-Zinn and Hanh, 2009; Tang et al. 2015). These interventions have shown positive effects on various components of EF, including attentional control, emotion regulation, and stress response (Tang, 2005, 2007, Tang et al. 2012; Tang and Posner 2009). Importantly, these effects are attributed specifically to the mindfulness components rather than mere contact with a trained interventionist.

Neuroimaging studies have demonstrated increased activation in the right dorsolateral and ventral lateral prefrontal cortex (PFC) following mindfulness training (Farb et al. 2007; Tang et al. 2015). Additionally, higher levels of mindfulness correlate with greater PFC activation (Xie et al. 2022; Lorenzetti et al. 2023; Yue et al. 2023), and experienced meditators exhibit enhanced PFC activity during various tasks (Hölzel et al. 2007). Functional magnetic resonance imaging (fMRI) studies further reveal that mindfulness training enhances performance on attentional control (Tang et al. 2015), IC (Jack et al. 2013), and WM tasks (Mrazek et al. 2013).

Despite these promising findings, a significant gap remains in the literature. Many studies have focused on the behavioral outcomes of mindfulness interventions without thoroughly investigating the underlying neural mechanisms, especially in young children. Moreover, while fMRI studies provide valuable insights, they are often limited by their constraints and less feasible with preschoolers. fNIRS offers a more portable and child-friendly alternative for neuroimaging, yet its application in mindfulness intervention studies remains underexplored.

Our study aims to address this gap by using fNIRS to examine the neural correlates of improved EF following mindfulness-based intervention in young children. By integrating multivariable and multiscale sample entropy analysis, we seek to provide a comprehensive understanding of how mindfulness training influences brain complexity and EF, offering novel insights that extend beyond current literature.

### Multivariate multiscale sample entropy

Entropy is a powerful tool for characterizing the regularity or complexity of a system (Elbert et al. 1994) and can be used to analyze systems at a single time scale (Costa et al. 2005). In the context of neuroimaging, Sample Entropy (SampEn) has been recognized as a biomarker for Alzheimer’s disease diagnosis when applied to fNIRS signaling in the frontal cortex (Li et al. 2018; Perpetuini et al. 2018, 2019). Additionally, SampEn has been used to study the effects of WM tasks on fNIRS signals (Angsuwatanakul et al. 2015).

Building on this, Ahmed and Mandic (2011) extended the concept of Multivariate Sample Entropy (MSE) by integrating it with the principles of multiscale entropy, resulting in Multivariate Multiscale Sample Entropy (MMSE). This innovative approach allows for the assessment of structural complexity across multiple time scales and variables, making it particularly suitable for analyzing the nonlinear dynamic behavior of the human brain (Zhu et al. 2020). MMSE has been successfully applied to various multichannel biomedical signals, such as electroencephalograms and fNIRS, providing deeper insights into the complex dynamics of these data (Ding et al. 2023).

Despite the demonstrated potential of MMSE in analyzing fNIRS signals, its application to the study of EF in children remains underdeveloped. Most existing research has focused on its use in diagnosing neurological conditions or assessing cognitive tasks in adults. The lack of studies employing MMSE to investigate the neural mechanisms underlying EF improvements in young children following mindfulness-based interventions represents a significant gap in the literature.

Our study aims to fill this gap by utilizing MMSE to analyze fNIRS data collected from young children undergoing mindfulness-based training. This approach will enable us to explore the complex neural dynamics associated with EF and assess the impact of mindfulness interventions on brain complexity. By doing so, we aim to provide novel insights into the neural underpinnings of EF development in early childhood, thereby advancing the field and addressing a critical gap in the current research landscape.

### The objective of this study

In the present study, we aimed to explore the effects of mindfulness-based training on children’s executive functioning and the changes in its neural mechanisms. Specifically, we administered a 15-session, twice per week mindfulness-based intervention to a group of 3- to 6-year-olds and used fNIRS to measure brain activity before and after the intervention, alongside MMSE analysis. This approach addresses the identified literature gap regarding the application of MMSE in studying EF in young children and the need for neuroimaging evidence to support the benefits of mindfulness interventions.

Based on this, this study proposed and examined the following two hypotheses:

**Hypothesis 1:** Children participating in the mindfulness-based intervention will demonstrate significant differences in executive functioning, as measured by the level of brain area activation, compared to pre-intervention baseline levels.

**Hypothesis 2:** After the mindfulness-based intervention, there will be a significant difference between the control and intervention groups in terms of accuracy and response time in the behavioral tasks and the level of activation of brain regions related to executive functioning, suggesting greater enhancement in the intervention group.

## Materials and methods

### Participants

This study recruited 112 typically developed preschool children. The parents of all the participating children provided written consent and were informed of the purpose of the study and the safety of the fNIRS experiment. The experiments were approved by the university ethics committee (PN-2021-038). Of these, 44 preschool children were excluded from the final analysis because of front-end or back-end fNIRS data tuning, poor data collection quality, or refusal to wear the cap. Ultimately, 68 children (*M*_age_ = 60.00 months, *SD* = 10.94) were included in the MMSE analysis. There was no statistically significant difference in the age of the participants between the intervention (*n* = 37, *M*_age_ = 60.03 months, *SD* = 11.14) and control (*n* = 31, *M*_age_ = 60.00 months, *SD* = 10.89) groups (*t* = 0.22, *P* = 0.98). The intervention and data collection occurred from October 2022 to June 2023.

### Measures

This study employed three tasks to assess EFs in children. The first task, Dimensional Change Card Sort (DCCS), is commonly used to study cognitive shifting in children. The second task, Go/No-Go, was used to measure IC in children. The third behavioral task was used to evaluate WM in children.

Stimuli were displayed on a computer screen, and the participants used a keyboard to respond. Before starting each task, the participants underwent training sessions to ensure that they understood the rules of the task. During the testing phase, the experimenter recorded each participant’s response time and actions, with participants clicking on the keyboard to make the corresponding responses. In the rest phases, participants were asked to look at a “+” on the computer screen and remain still.

#### DCCS task

The DCCS task is commonly used to assess children’s CF, also known as task-switching or set-shifting. This experiment, designed by Zelazo’s (2006) team, has been widely applied in studying the development of EF during early childhood. In the traditional DCCS task, two target cards are displayed on a computer screen, such as a blue rabbit and a red boat, and participants are required to categorize a series of cards that have two attributes. These stimulus cards possess two dimensions: color and shape. The target cards (like a red boat and a blue rabbit) match the test cards (such as a blue boat or a red rabbit) on one dimension but not on the other.

**Fig. 1:**
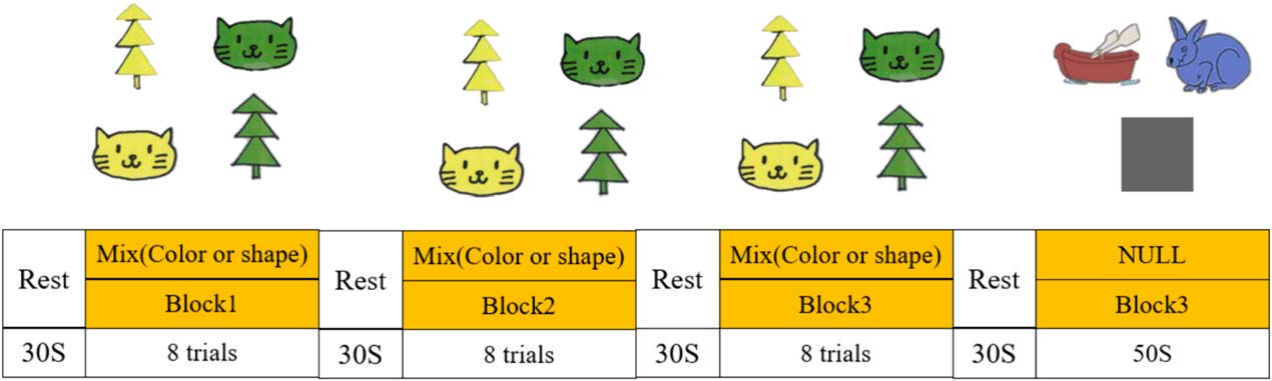
Task paradigm of the DCCS task.

Based on Moriguchi et al’.s (Moriguchi et al. 2007; Moriguchi and Itakura 2008) modification and our previous study (Xie et al. 2022), the present study further modified the DCCS task by introducing a mixed sorting card task to increase the task difficulty and a blank card task to exclude the effects from keystrokes (Fig. 1). In the mixed sorting card task phase, participants were asked to sort test cards based on verbal instructions (shape or color). In the blank card task phase, participants were asked to randomize key presses to eliminate the effects of key press actions on the brain. The experiment consisted of four consecutive test blocks interspersed with four rest phases. During the rest phases, a “+” symbol appeared in the center of the screen and participants were instructed to focus on the symbol and remain still. The first three test blocks were mixed blocks containing eight randomized trials. The fourth test block was a blank task lasting 50 s. Participants responded by pressing keys on the keyboard, and the computer recorded the answers and reaction times for the tests. The total number of correct answers in each block was calculated as an indicator of overall performance for subsequent analysis.

#### Go/No-Go Task

**Fig. 2:**
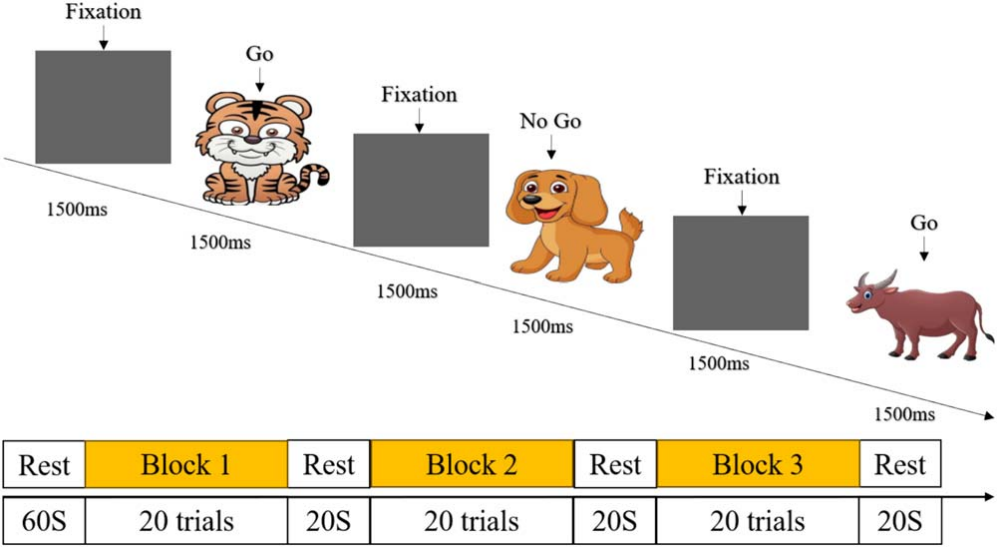
Task paradigm of the Go/No-Go task.

The Go/No-Go task, modified from the paradigm of Lahat et al. (2010) and our previous study (Xie et al. 2022) by replacing the fixation picture with the same picture as the screen background to reduce interference, aims to measure children’s IC. This task was chosen for its proven effectiveness and solid neural basis mapping (Wiebe et al. 2012). In each trial (Fig. 2), images of animals (such as cows, horses, tigers, or dogs) appear on the screen. The participants were instructed to press the space bar on the keyboard immediately upon seeing any animal except for a dog (Go stimulus) and not to press when a dog image appeared (No-Go stimulus). During the practice phase, there were four Go trials and four No-Go trials, with participants receiving reminders when they made incorrect responses. The entire task was divided into three blocks, each containing 16 Go trials and 4 No-Go trials, which were randomly assigned. Participants had resting periods after completing each block of trials. For subsequent analysis, accuracy was calculated by dividing the number of correct responses by the total number of trials.

#### WM task

**Fig. 3:**
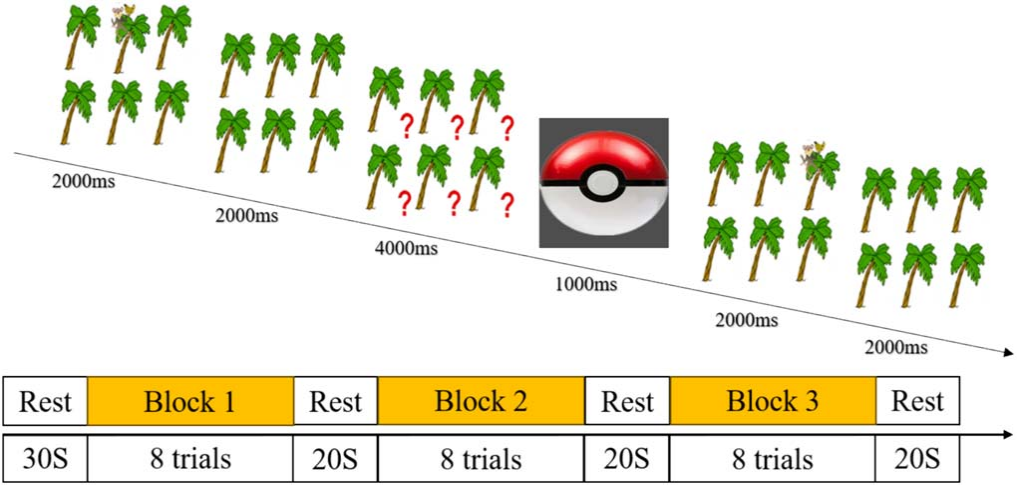
Task paradigm of the WM task.

The third behavioral task is an adaptation from a previous study (Perlman et al. 2016) designed to measure the WM capacity of preschool children aged 3–6 years. In this experiment (Fig. 3), each trial displayed six trees on the screen, with a monkey randomly appearing on one of the six trees. Participants were required to remember the location where the monkey appeared. After 2 s, the monkey disappeared, leaving the image of the six trees on display for another 2 s. Subsequently, a question mark appeared at the location of each tree on the screen, and the participants had 4 s to indicate the monkey’s previous position. The experimenter recorded the answer by pressing the corresponding key on the basis of the participant’s indication. During the practice phase, participants received feedback that indicated whether they responded correctly or not. The entire task was divided into three blocks, each containing eight trials. Between each trial, an unrelated image appeared for 1 s to remind participants to prepare for the next trial. There was a 20-s rest period for participants between the three blocks of the task. For subsequent analysis, accuracy was calculated by dividing the number of times the correct location was indicated by the total number of trials.

### fNIRS data recording and processing

**Fig. 4:**
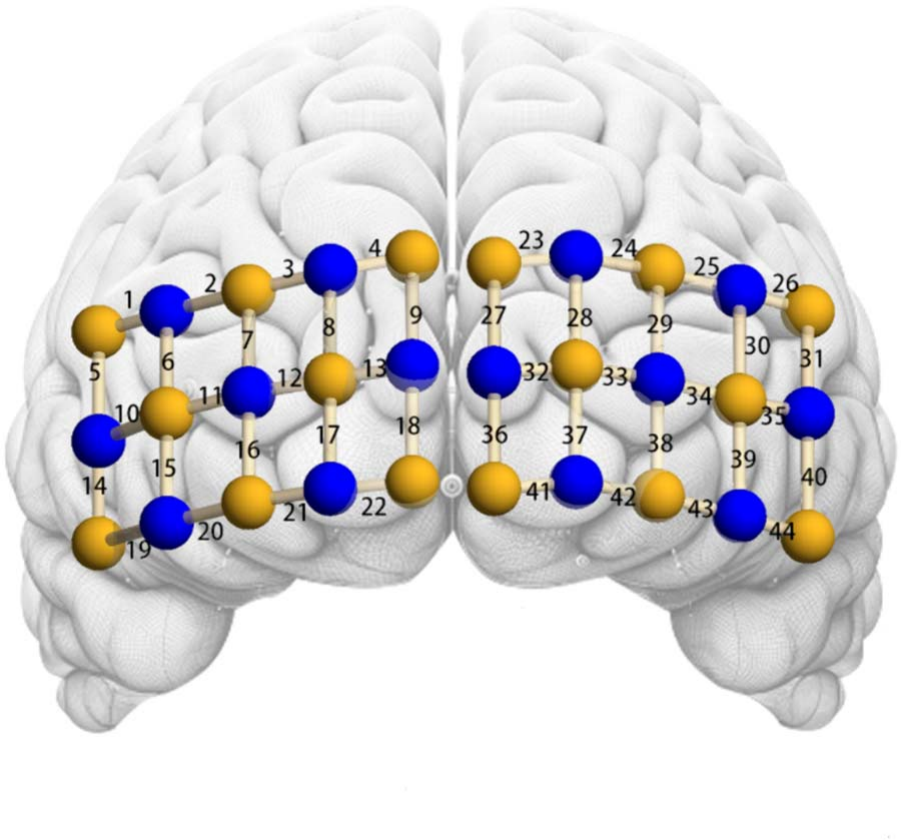
Localization of regions of interest.

During cognitive tasks, brain regions that are more active require more oxygen, leading to an increase in oxygenated hemoglobin and a decrease in deoxygenated hemoglobin in these areas. fNIRS (Ferrari and Quaresima 2012) is a non-invasive imaging technique used to monitor brain activity. It indicates neural activity by measuring changes in cerebral blood flow, specifically the levels of oxygenated and deoxygenated hemoglobin. In this study, a multi-channel fNIRS system (OxyMon MK III, Artinis, The Netherlands) was used to measure the concentration changes of oxygenated hemoglobin (HbO) and deoxygenated hemoglobin (HbR) in participants during the experiment. Based on EF research in children (Diamond 2013) and the experimental design of the current study, we employed a 3 × 10 light pole template located at the forehead, covering the frontal area with 44 channels. To ensure consistent light pole array placement for all participants, the central-lower position of the array was located at the Fpz position, consistent with the 10-20 measurement system. Accordingly, the regions of interest (ROIs) were the left ventrolateral prefrontal cortex (VLPFC), right VLPFC, left dorsolateral prefrontal cortex (DLPFC), right DLPFC, left posterior superior frontal cortex (PSFC), right PSFC, left temporal cortex (TC), right TC, and medial prefrontal cortex (MPFC) (Fig. 4). Previous studies have shown (Menon and D’Esposito 2022; Buckner and DiNicola 2019) that the frontal regions are actively involved in the maintenance and regulation of EF. The sampling rate was set at 50 Hz for data collection. The differential pathlength factor (DPF) value for each participant was calculated based on their age using the formula (DPF = 4.99 + 0.0678 × Age^0.814).

### Procedure

First, the principal of the target preschool was contacted and explained of the study purpose. Then, print questionnaires and consent forms were enveloped and carried home by the children to their parents, who gave consent and filled the questionnaires. The children then brought them back to the preschool, and class teachers collected these forms and passed them to the research team. Next, the children were invited to complete the three EF tasks in a quiet classroom in the preschool. Before the tasks, an experienced NIRS technician placed the child-sized NIRS cap and installed the optodes. At the same time, a research assistant who majored in early childhood education engaged in story-book reading with the child. All three tasks were computerized using Psychophysics Toolbox extensions and displayed on a 52.71 cm × 29.65 cm Huawei monitor. The children were trained to perform the tasks before each experiment began. The order of the three tasks was randomly assigned.

After the pre-intervention data collection, the participants were randomly assigned to the intervention and control groups. This mindfulness intervention was adapted from the MindUP training package to be both playful and mindful for preschoolers. A certified MindUP trainer and a group of researchers in early childhood education adapted the training course. The class teachers of the intervention groups received training from the certified teacher and delivered mindfulness training during the school day. The interventions were structured in three parts: my mindful brain, my five senses, and my mindful world. For the intervention group, the children received 20 min per session, twice a week, all together 15 sessions of mindfulness intervention. After the intervention, all participants from both the intervention and control groups were assessed for their EF again.

### Data analysis plan

#### Behavioral results analysis

In Matlab, we analyzed the behavioral results of the participants in the three experimental tasks. Specifically, for the DCCS and GO/NO-GO tasks, we calculated the response time and accuracy for each participant, while for the WM task, only accuracy was calculated. Then, because the behavioral data did not fit a normal distribution, we used Wilcoxon Signed-Rank Test to compare the differences before and after the mindfulness-based training as well as the differences in the intervention group before and after the intervention in order to explore whether the changes in the brain manifested in the behavioral data. All of our statistics use the false discovery rate correction.

#### fNIRS data preprocessing

For preprocessing of fNIRS data, we used the Homer2 (Huppert et al. 2009) software package to process the multi-channel near-infrared data. First, we manually removed channels with poor quality or bad contact to ensure data quality, converted raw signals into optical density signals, and applied a wavelet transform algorithm (Molavi and Dumont 2012) to remove motion artifacts. These steps helped reduce signal interference caused by participant movement. Next, we filtered the data using a third-order Butterworth filter to remove frequencies outside the range of 0.01–0.08 Hz. This aided in reducing high-frequency noise and low-frequency drift. We then converted optical density signals into concentration changes of oxygenated and deoxygenated hemoglobin and conducted correlation-based signal improvement to further correct for motion artifacts (Engelmann et al. 2012).

#### Statistical analysis

We performed MMSE calculations on the preprocessed fNIRS signals to measure brain complexity. MMSE is an extension based on the MSE. MSE is used to calculate the complexity of a single time series at different temporal scales.

The calculation of the MMSE is divided into two steps:

Coarse-Graining of Multivariate Time Series: the multivariate time series ${\left\{{x}_k,i\right\}}_{i=1}^N$, where $k=1,2,\dots, p$, and $p$ represents the number of variables (channels), and$N$ is the number of samples per variable, is coarse-grained to define increasing time scales. For a scale factor$\tau$, the coarse-graining process comes in two forms: non-overlapping, where every $\tau$ data points are jumped and averaged to produce a new data point; and overlapping, where the jump is 1 − $\tau$ data points, averaging $\tau$ data points each time (Busa and Van Emmerik 2016). The mathematical definition of a non-overlapping multivariate coarse-grained time series is as follows:


$$ {y}_{j,k}^{\left(\tau \right)}=\frac{1}{\tau}\sum_{i=\left(j-1\right)\tau +1}^{j\tau}{x}_{i,k,} $$

where $1\le j\le \frac{N}{\tau }$ and $k=1,\dots, p$.

Coarse-grain the original multivariate time series and calculate the MSE for each coarse-grained sequence. Plot the relationship between MSampEn and the scale factor $\tau$.

MSE is the calculation of multiscale entropy simultaneously across multiple channels. For a $p$-variate time series ${\left\{{x}_k,i\right\}}_{\mathrm{i}=1}^{\mathrm{N}}$, where $k=1,2,\dots, p$, the mathematical definition of MSE is:

Construct $\left(N-n\right)$ composite delay vectors ${X}_m(i)\in{R}^{\mathrm{m}}$, where $i=1,2,\dots, N-n$, and $n=\mathit{\max}\left\{M\right\}\times \mathit{\max}\left\{\tau \right\}$.

Define the distance $d$[${\mathrm{X}}_m\left(\mathrm{i}\right),{\mathrm{X}}_m\left(\mathrm{j}\right)$ between any two composite delay vectors ${\mathrm{X}}_m\left(\mathrm{i}\right)$ and ${\mathrm{X}}_m\left(\mathrm{j}\right)$ as the absolute value of the largest difference between their corresponding elements. That is:


(1) \begin{equation*} \mathrm{d}\left[{X}_m(i),{X}_m(j)\right]={\mathit{\max}}_{k=0,\dots, m-1}\left(\left|x\left(i+k\right)-x\left(j+k\right)\right|\right). \end{equation*}

For a given ${X}_m(i)$ and threshold $r$, count the number of instances when the distance between ${X}_m(i)$ and ${X}_m(j)$ is less than or equal to $r$ (where $\left(1\le j\le N-n,j\ne i\right)$), denoted as ${P}_i$. Then, calculate the frequency of occurrence: 


(2) \begin{equation*} {B}_i^m(r)=\frac{1}{N-n-1}{P}_i, \end{equation*}

And define a global quantity: 


(3) \begin{equation*} {B}^m(r)=\frac{1}{N-n}\sum_{i=1}^{N-n}{B}_i^m(r). \end{equation*}

Increase the dimension to $m+1$ and calculate the number of instances where the distance between ${X}_{m+1}(i)$ and ${X}_{m+1}(j)$ (for $1\le j\le N-n,j\ne i$) is less than or equal to $r$, denoted as ${A}_i$. Then, compute the frequency of occurrence: 


(4) \begin{equation*} {B}_i^m(r)=\frac{1}{p\left(N-n\right)-1}{Q}_i, \end{equation*}

And define a global quantity: 


(5) \begin{equation*} {B}^{m+1}(r)=\frac{1}{p\left(N-n\right)}\sum_{i=1}^{p\left(N-n\right)}{B}_i^{m+1}(r). \end{equation*}

Thus, $ {B}^m(r) $ is the probability of any two composite delay sequences matching at $ m $ points under the similarity tolerance $ r $, and $ {B}^{m+1}(r) $ is the probability of any two composite delay sequences matching at $ m+1 $ points.

For a tolerance level $r$, Multivariate Sample Entropy (MSamEn) is calculated as the negative natural logarithm of the conditional probability that two composite delay vectors, which are close to each other in an $m$-dimensional space, remain close when the dimension is increased by 1. The calculation formula is as follows: 


(6) \begin{equation*} M\ SampEn\left(M,\tau, r,N\right)=-\mathit{\ln}\left[\frac{B^{m+1}(r)}{B^m(r)}\right]. \end{equation*}

Multivariate Sample Entropy (MSampEn) adopts the algorithm by Cao (Ahmed and Mandic 2011). Regarding the choice of $r$, it is important to remember that to avoid significant contributions from noise to the sample estimation, $r$ must be larger than the noise level present in most of the signal. Another criterion for selecting $r$ is based on the dynamic characteristics of the signal (signal dynamics) (Ahmed and Mandic 2012). Typically, $r$ is generally taken as $0.15\ast std\left({x}_k\right)$. The scale factor $\tau$ is calculated using the autocorrelation function (Cao 1997).

Next, we divided the 44 fNIRS channels into nine ROIs, including the left VLPFC, right VLPFC, left DLPFC, right DLPFC, left PSFC, right PSFC, left TC, right TC, and MPFC. Our aim was to investigate whether there were significant differences in the complexity of these ROIs across different tasks.

All calculations were analyzed by *t*-test using SPSS software. In the preliminary analysis, we compared the complexity of each ROI before and after mindfulness training using paired t-tests to assess the impact of mindfulness training on brain regions in the intervention group (Hypothesis 1). In processing the fNIRS data from the DCCS task, we implemented an innovative methodology by calculating the difference between the fNIRS data from each task phase and that from a null task (random key presses). This approach effectively eliminated neural activity unrelated to the task, thus more accurately reflecting brain activity changes induced by specific tasks. This finding is significant because it validates the precision of the adopted method in capturing neurophysiological changes triggered by task engagement. We also compared the complexity of each ROI before and after mindfulness training using paired t-tests to assess the impact of mindfulness training on brain regions in the control group. In a more in-depth analysis, we calculated the difference in the MMSE results and behavioral performance of the participants before and after training. We then compared the differences between the intervention and control groups using t-tests to explore the specific effects of positive thinking training on the brain (Hypothesis 2). All of our statistics use the FDR correction。.

## Results

**Fig. 5:**
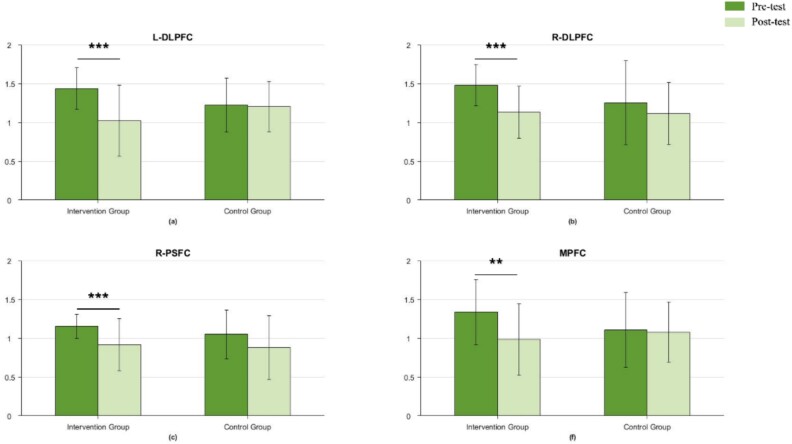
Comparison of MMSE before and after the intervention for the DCCS task (full sample).

The *t*-test analysis was conducted on the MMSE results of the intervention and control groups before and after the tests. The MMSE comparison results for the DCCS task are shown in Fig. 5. In the DCCS task, significant differences were observed in the intervention group in the left DLPFC (*t* = 4.713, *P* < 0.001) (Fig. 5a), right DLPFC (*t* = 4.964, *P* < 0.001) (Fig. 5b), right PSFC (*t* =3.741, *P* < 0.001) (Fig. 5c), and MPFC (*t* =3.262, *P* < 0.01) (Fig. 5d), but no significant changes in the control group.

**Fig. 6:**
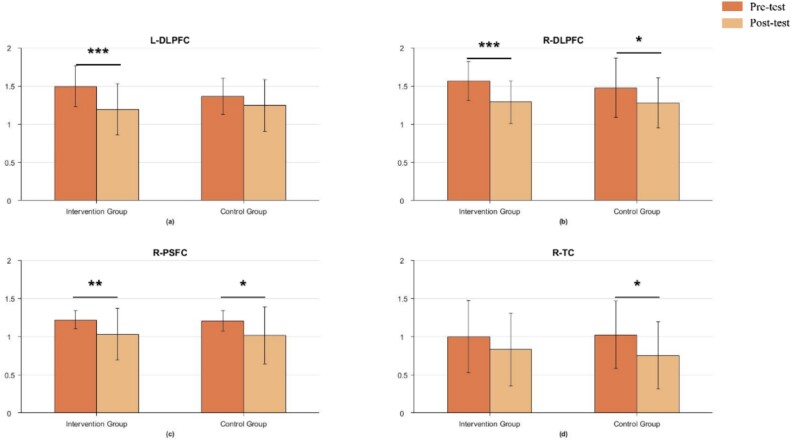
Comparison of MMSE before and after the intervention for the Go/No-Go task (full sample).

The MMSE comparison results for the GO/NO-GO task are shown in Fig. 6. In the intervention group during the GO/NO-GO task, significant differences were observed in the left DLPFC (*t* = 4.348, *P* < 0.001) (Fig. 6a), right DLPFC (*t* = 44.477, *P* < 0.001) (Fig. 6b), and right PSFC (*t* = 3.079, *P* < 0.01) (Fig. 6c). Significant differences were also observed in the control group in the right PSFC (*t* = 2.147, *P* < 0.05) (Fig. 6c) and right TC (*t* = 2.132, *P* < 0.05) (Fig. 6d).

**Fig. 7:**
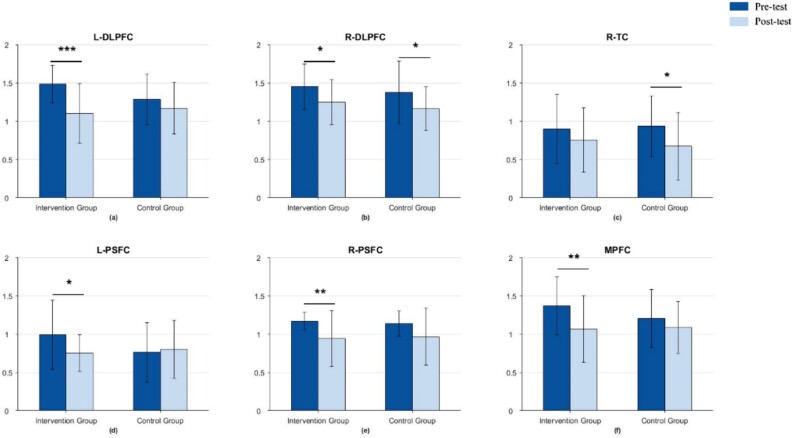
Comparison of MMSE before and after the intervention for the WM task (full sample).

The MMSE comparison results for the WM task are shown in Fig. 7. In the intervention group during the WM task, significant differences were observed in the left DLPFC (*t* = 4.453, *P* < 0.001) (Fig. 7a), right DLPFC (*t* = 2.662, *P* < 0.05) (Fig. 7b), left PSFC (*t* = 2.906, *P* < 0.05) (Fig. 7d), right PSFC (*t* = 3.125, *P* < 0.01) (Fig. 7e), and MPFC (*t* = 2.697, *P* < 0.01) (Fig. 7f). Significant differences were also observed in the control group in the right DLPFC (*t* = 2.301, *P* < 0.05) (Fig. 7b) and right TC (*t* = 2.140, *P* < 0.05) (Fig. 7c).

Notably, in all brain regions where significant differences were observed, there was a trend of decreased complexity (i.e. post-test MMSE results were lower than pre-test), indicating that the complexity of relevant brain regions decreased after mindfulness-based training. Putting together, the findings indicated that mindfulness-based intervention demonstrated significant differences in executive functioning, as measured by the level of brain area activation, compared to pre-intervention baseline levels, supporting H1.

**Fig. 8:**
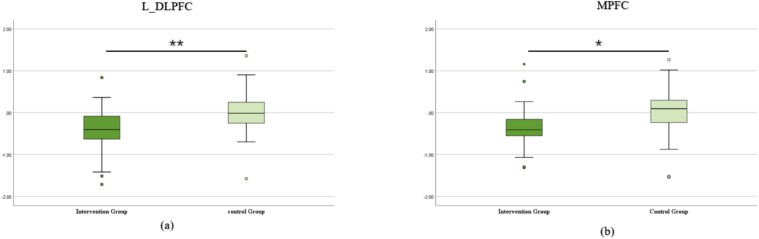
Pre-test and post-test difference comparison between intervention and control groups for the DCCS task.

More direct evidence of the positive effect of mindfulness training came from difference comparison in MMSE and behavioral results between the intervention and control groups. The difference comparison results for the DCCS task are presented in Fig. 8. For the DCCS task, significant differences were observed between the intervention and control groups in the left DLPFC (*t* = − 3.178, *P* < 0.01) and MPFC (*t* = 2.232, *P* < 0.05). These results suggest that mindfulness training had a significant impact on the activity of the left DLPFC and MPFC in the DCCS task. Appendix 1 displayed the results of difference comparison for all ROIs for the DCCS task.

The difference comparison results for the GO/NO-GO task showed no significant differences were observed during the GO/NO-GO task (*P* < 0.05), suggesting that mindfulness training might not have differentially affected brain activity between the intervention and control groups. Appendix 2 displayed the results of difference comparison for all ROIs in the GO/NO-GO task.

**Fig. 9:**
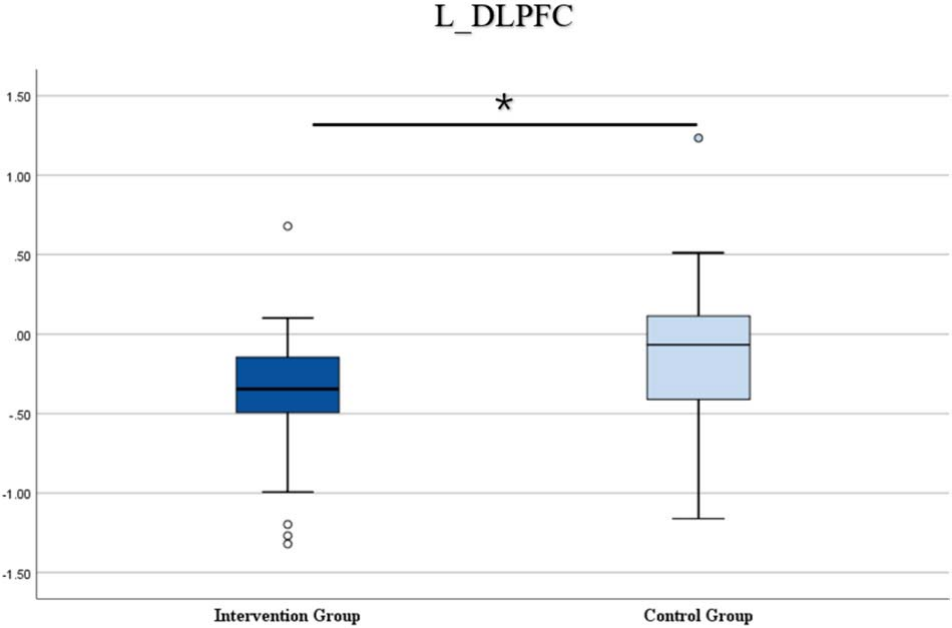
Pre-test and post-test difference comparison between intervention and control groups for the WM task.

The difference comparison results for the WM task are also displayed in Fig. 9. During the task, a significant difference was observed in the left DLPFC (*t* = − 2.426, *P* < 0.05), indicating that mindfulness training significantly impacted the activity of the left DLPFC in the WM task. Appendix 3 displayed the results of difference comparison for all ROIs in the WM task.

**Fig. 10:**
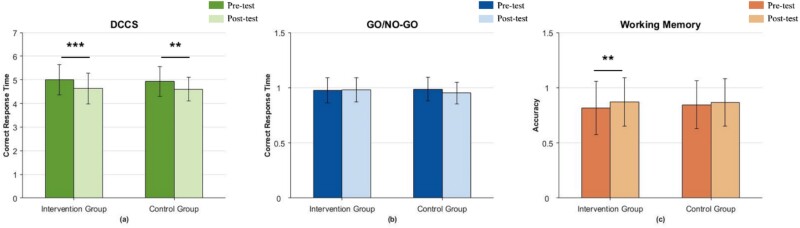
Comparison of behavioral data between pre- and post-tests.

The results of the different comparisons of the pre- and post-tests of the behavioral data are shown in Fig. 10. In the DCCS task (Fig. 10a), there was a significant difference in correct response time for both the intervention (*Z* = − 3.674, *P* < 0.001) and control groups (*Z* = − 2.841, *P* < 0.01), but the change was more pronounced in the intervention group. In the GO/NO-GO task (Fig. 10b), there were no differences in either group. This may indicate that although some changes occurred at the neurophysiological level, these changes may not yet be reflected at the behavioral level, or the task design may not have been effective in capturing changes at the behavioral level. In the WM task (Fig. 10(c), the intervention group ($Z$=2.594, *P* < 0.01) showed a significant increase in correctness. Putting together, significant differences between the control and intervention groups in terms of the level of activation of brain regions related to executive functioning and behavioral tasks were observed, with greater enhancement in the intervention group, thus supporting H2.

## Discussion

In this study, we found MMSE changes before and after mindfulness training in all three EF tasks (H1) and confirmed the positive effects of mindfulness training on children’s brain complexity and on the promotion of EF in specific brain regions (H2). We will discuss these findings in details in the following section.

### Decreased brain complexity after intervention

The current study found significant decreases in MMSE in the LDLPFC, RDLPFC, RPSFC, and MPFC during the DCCS task, decreases in the LDLPFC, RDLPFC, RPSFC, and RTC during the GO/NO-GO task, as well as decreases in the LDLPFC, RDLPFC, LPSFC, RPSFC, and MPFC during the WM task, in the intervention group. From an informational perspective, fNIRS is a non-smooth, non-linear time series, so its entropy can be used to distinguish between different healthy brain states (Ghouse et al. 2020). Based on this, MMSE is proposed based on sample entropy, as a measure of time series complexity, with smaller the entropy, smaller the time series complexity and higher the self-similarity (Wang et al. 2021). In children, the decrease in complexity of brain regions may be related to different stages of brain development. During early childhood, the brain undergoes a phase of rapid development and shaping, which involves the formation of a large number of neurons and synapses, as well as the establishment and remodeling of neural networks (Gilmore et al. 2018; McLaughlin et al. 2019). During this process, brain regions may exhibit relatively complex structures and functions in response to various learning and cognitive tasks. Thus, the high complexity of the brain implies that more cognitive functions are involved (Ding et al. (2023).

However, as children grow, the brain progressively optimizes and consolidates these connections and may have fewer redundant connections or functions in specific brain regions (McLaughlin et al. 2014; Cao et al. 2017). This reduction in brain region complexity may reflect the brain’s streamlining and optimisation during learning and development, allowing it to perform tasks more efficiently. This phenomenon may play an important role in the development of a number of cognitive abilities, such as improved concentration, memory and problem-solving skills. A decrease in brain complexity, as measured by entropy, might reflect this increased efficiency and reduced neural variability. Previous research suggests that higher entropy in neural signals can indicate a more disorganized and less efficient brain state (Li et al. 2018). Therefore, the observed reduction in complexity could signify a shift towards more streamlined and effective neural processing in the DLPFC and PSFC, regions crucial for EF.

Furthermore, MMSE was able to discover specific brain region activity in oxygenated hemoglobin that could not be resolved using mean estimates of any of the three hemoglobin concentrations (HbR, HbO, HbT). This enhanced sensitivity may stem from the additional quantification of nonlinear and complex kinetic properties provided by entropy analysis. Even when linear effects are weak or insignificant, nonlinear and complex behavioral patterns may remain and can be detected (Perpetuini et al. 2020; Zhang et al. 2021). Thus, the MMSE method is more sensitive in identifying changes in brain regions and more effective in revealing the effects of interventions compared with traditional methods. When the entropy of brain activity decreases (complexity decreases), this could mean that the activity in the brain network becomes more sequential or that the way the brain processes information becomes more efficient and focused. Thus, the reduction in complexity might also reflect the optimization of neural resources. As children become more adept at mindfulness practices, their brains may require fewer resources to maintain focused attention and executive control, leading to decreased variability in neural signals.

In contrast, although the control group also showed reduced complexity of brain regions in the WM and GO/NO-GO tasks, the degree and extent of change was not as significant as in the intervention group. This situation may be due to the fact that even though the children were not trained in mindfulness, they experienced the development of brain functioning over a two-month period, resulting in differential changes in the pre- and post-tests.

### Positive effects of mindfulness training on cognitive functioning

To further explore the positive effects of mindfulness training on children’s cognitive functioning, brain complexity differences between the intervention and control groups before and after mindfulness training was examined. It was found that mindfulness training showed a positive effect on the performance of the DCCS and WM tasks, particularly significantly in the left DLPFC. Previous research has shown that the left DLPFC develops more rapidly during early childhood, when language skills develop rapidly. This development is critical for tasks involving verbal WM and logical reasoning. On the other hand, the right DLPFC continues to develop throughout childhood, supporting the maturation of spatial WM, attentional control, and emotion regulation (Goto et al. 2015). Our study provides evidence that mindfulness-based training has a positive effect on the development of CF and WM.

According to the IAA model (Shapiro et al. 2006) and the view of attentional modulation as a core component of the mindfulness mechanism (Tang et al. 2015), mindfulness training works by enhancing sustained attention, better monitoring, and effective switching between task sets (i.e. CF; Vieth and von Stockhausen 2022). The results of the present study are consistent with previous research showing that mindfulness training stabilizes attention and improves CF (Lee and Orsillo 2014; Zou et al. 2020). Individuals typically activate the DLPFC and parietal cortex during the early stages of mindfulness training, a phenomenon that supports the findings of studies conducted with preschoolers who are new to mindfulness training (Posner et al. 2015; Tang et al. 2015).

Furthermore, the current study showed that children in the mindfulness training group showed improved number of consecutive correct responses compared to the control group, suggesting that mindfulness interventions can promote sustained accuracy in WM tasks. This trend may imply that mindfulness training helps to reduce the stress-induced brain load in WM tasks, which in turn reduces the intensity of brain activity. This finding corroborates with a study by Jha et al. (2010), which found that mindfulness training was effective in reducing stress levels and facilitating better performance on a WM task. Similarly, Zeidan et al. (2010) explored the effects of short-term mindfulness training relative to an active control group (i.e. listening to an audiobook) on performance on an N-back task. It is worth noting that Mrazek et al. (2013) also found that mindfulness training was able to reduce distraction during the performance of a WM task. These about findings suggest that mindfulness training may reduce brain activity during WM tasks by reducing the burden of distracting thoughts.

However, similar behavioral and neural results were not observed in the Go/No-Go task, suggesting that mindfulness training may not have significantly improved children’s IC, although a large body of research has shown that the practice of mindfulness meditation can significantly enhance individuals’ IC on both behavioral and neural levels (Allen et al. 2012; Heeren et al. 2009; Moore and Malinowski 2009; Sahdra et al. 2011; Teper et al. 2013). This non-significant change may stem from design issues with the Go to No-Go ratio in the current study and a previous study (Xie et al. 2022), limiting children’s opportunities to perform on No-Go trials, which are typically associated with higher levels of mindfulness (Logemann-Molnár et al. 2022). Another reason might be that the spatiotemporal dynamics of IC change with age (Knežević and Marinković, 2017; Lin et al. 2018). Thus, IC is a long-term developmental function that requires longer interventions. Future research should conduct further studies using time-sensitive methods across the lifespan, exploring in more detail the methods of task design and behavioral outcome assessment to gain a more comprehensive understanding of the combined effects of cognitive training on the brain and behavior. Future research should examine in greater depth the role of these changes in actual behavioral performance, as well as their potential benefits for improving specific cognitive dysfunctions such as attention deficits.

Finally, the use of fNIRS in this study provided valuable insights into the task-related changes in cerebral hemodynamics associated with mindfulness-based training in young children. However, it is important to acknowledge the limitations inherent in interpreting fNIRS data as direct indicators of underlying neurophysiological or functional changes in cortical organization. While the findings suggest alterations in brain activation patterns following the intervention, caution must be exercised when extrapolating these hemodynamic responses to infer specific changes in cortical function or organization. fNIRS primarily measures changes in cerebral blood flow and oxygenation, which are influenced by various factors, including vascular dynamics, systemic physiological changes, and motion artifacts (Scholkmann et al. 2014). As such, observed hemodynamic responses may not always reflect direct neural activity changes and should be interpreted with caution. Furthermore, the spatial resolution of fNIRS is limited compared to other neuroimaging techniques such as fMRI (Boas et al. 2014). This limitation may restrict our ability to precisely localize brain activation changes and infer their neuroanatomical correlates. Therefore, future studies could benefit from integrating fNIRS data with complementary neuroimaging modalities to provide a more comprehensive understanding of the neural mechanisms underlying mindfulness-based interventions (Eggebrecht et al. 2014).

## Conclusion

Although our study demonstrated that the intervention had an effect on executive functioning, there were many factors involved (e.g. age, gender, IQ, etc.). In future studies, we should categorize children by age and gender and investigate whether mindfulness training has different improvement effects at different ages and genders. Further, we can explore whether mindfulness training is more effective in children with high IQ. In studying the development of executive functioning in children, we need to realize that children respond in a variety of ways; therefore, tasks to assess executive functioning should reflect this diversity. In particular, in the go/No-Go task, the present study failed to observe a significant effect of mindfulness training, which may be related to problems with the task design and children’s tendency to become tired and have difficulty sustaining their concentration during the task. Future studies should appropriately increase the proportion of No-Go trials and consider toing children’s interest and concentration. Follow-up studies should also determine whether these interventions are effective in increasing inhibitory efficiency and improving cognitive domains mediated by IC. Stronger evidence for the improvement of brain function by mindfulness-based training in the other two tasks is needed. Further exploration is needed to determine what factors may influence the effectiveness of mindfulness interventions. Most of the current studies vary widely in the duration of the intervention; therefore, determining the optimal amount of mindfulness practice is important for studies that promote executive functioning (or other outcomes of interest).

Another limitation of this study is the reliance on fNIRS as the primary neuroimaging modality. While fNIRS offers several advantages, including portability and fewer participant constraints compared to fMRI, it is essential to acknowledge its limitations in accurately capturing the complex dynamics of cortical function. The study’s interpretation of observed hemodynamic changes as indicative of real neurophysiological or functional changes in cortical organization should be approached with caution. Future research should aim to validate fNIRS findings using complementary techniques and consider alternative methods for investigating cortical organization changes more directly.

The current study used a new method to reveal changes in brain regions by calculating the entropy of fNIRS data during the experiment. It was found that entropy reveals areas that produce neuronal correlations and is consistent with the results of traditional methods of analyzing neuronal correlations, as well as providing information not found in mean estimates. The current study found that 15-session of mindfulness training enhanced CF and WM in young children. These brain region changes in EF were evidenced by significant decreases in brain complexity during CF and WM tasks, suggesting that preschoolers can benefit from mindfulness training. Research on EF interventions in schools is in its infancy, and future studies should include generalization and optimal developmental period factors to maximize intervention effects.

## Supplementary Material

Supplementary_bhae330
