# Supplementary material for: Using mindfulness-based intervention to promote executive function in young children: a multivariable and multiscale sample entropy study
Source: Cereb Cortex. 2024 Sep 5;34(9):bhae330. doi: 10.1093/cercor/bhae330 (PMC11375865; doi:10.1093/cercor/bhae330)
Supplement: Supplementary_bhae330 [file supplementary_bhae330.docx]

*Appendix 1.* Pre-test and post-test difference comparison between intervention and control groups for the DCCS task.

| DCCS | Intervention VS Control Group | Mean | *t*-value | *p*-value |
| --- | --- | --- | --- | --- |
| L-VLPFC | Intervention Test | -0.213 | -1.111 | 0.287 |
|  | Control Group | 0.135 |  |  |
| R-VLPFC | Intervention Group | -0.044 | 0.254 | 0.803 |
|  | Control Group | -0.086 |  |  |
| L-DLPFC | Intervention Group | -0.429 | -3.178 | 0.001** |
|  | Control Group | -0.019 |  |  |
| R-DLPFC | Intervention Group | -0.362 | -1.789 | 0.080 |
|  | Control Group | -0.139 |  |  |
| L-PSFC | Intervention Group | -0.342 | -1.004 | 0.372 |
|  | Control Group | 0.000 |  |  |
| R-PSFC | Intervention Group | -0.242 | -0.481 | 0.634 |
|  | Control Group | -0.181 |  |  |
| L-TC | Intervention Group | -0.359 | -1.016 | 0.367 |
|  | Control Group | 0.002 |  |  |
| R-TC | Intervention Group | -0.109 | -0.225 | 0.823 |
|  | Control Group | -0.059 |  |  |
| MPFC | Intervention Group | -0.322 | -2.232 | 0.020* |
|  | Control Group | 0.001 |  |  |
| **p* < 0.05; ***p* < 0.01; ****p* < 0.001. | | | |  |

*Appendix 2.* Pre-test and post-test difference comparison between intervention and control groups for the Go/No-Go task.

| GO/NO-GO | Intervention VS Control Group | Mean | *t*-value | *p*-value |
| --- | --- | --- | --- | --- |
| L-VLPFC | Intervention Test | -0.010 | 0.316 | 0.758 |
|  | Control Group | -0.120 |  |  |
| R-VLPFC | Intervention Group | -0.084 | -0.244 | 0.810 |
|  | Control Group | -0.033 |  |  |
| L-DLPFC | Intervention Group | -0.329 | -1.958 | 0.055 |
|  | Control Group | -0.115 |  |  |
| R-DLPFC | Intervention Group | -0.276 | -0.721 | 0.474 |
|  | Control Group | -0.206 |  |  |
| L-PSFC | Intervention Group | -0.236 | -0.189 | 0.859 |
|  | Control Group | -0.180 |  |  |
| R-PSFC | Intervention Group | -0.236 | -0.189 | 0.859 |
|  | Control Group | -0.180 |  |  |
| L-TC | Intervention Group | -0.539 | -1.481 | 0.199 |
|  | Control Group | -0.001 |  |  |
| R-TC | Intervention Group | -0.053 | 0.614 | 0.543 |
|  | Control Group | -0.181 |  |  |
| MPFC | Intervention Group | -0.145 | -0.215 | 0.831 |
|  | Control Group | -0.113 |  |  |
| **p* < 0.05; ***p* < 0.01; ****p* < 0.001. | | | |  |

*Appendix 3.* Pre-test and post-test difference comparison between intervention and control groups for the Working Memory task.

| Working Memory | Intervention VS Control Group | Mean | *t*-value | *p*-value |
| --- | --- | --- | --- | --- |
| L-VLPFC | Intervention Test | -0.219 | 0.095 | 0.927 |
|  | Control Group | -0.262 |  |  |
| R-VLPFC | Intervention Group | 0.066 | 0.310 | 0.761 |
|  | Control Group | 0.003 |  |  |
| L-DLPFC | Intervention Group | -0.409 | -2.426 | 0.019* |
|  | Control Group | -0.114 |  |  |
| R-DLPFC | Intervention Group | -0.240 | -0.283 | 0.778 |
|  | Control Group | -0.213 |  |  |
| L-PSFC | Intervention Group | -0.326 | -0.516 | 0.643 |
|  | Control Group | -0.139 |  |  |
| R-PSFC | Intervention Group | -0.326 | -0.516 | 0.643 |
|  | Control Group | -0.139 |  |  |
| L-TC | Intervention Group | -0.353 | -0.999 | 0.374 |
|  | Control Group | -0.005 |  |  |
| R-TC | Intervention Group | -0.137 | 0.316 | 0.754 |
|  | Control Group | -0.210 |  |  |
| MPFC | Intervention Group | -0.263 | -1.015 | 0.315 |
|  | Control Group | -0.093 |  |  |
| **p* < 0.05; ***p* < 0.01; ****p* < 0.001. | | | |  |
